# Supplementary material for: S100A4 exerts robust mucosal adjuvant activity for co-administered antigens in mice
Source: Mucosal Immunol. 2022 Jun 21;15(5):1028–39. doi: 10.1038/s41385-022-00535-6 (PMC9212208; doi:10.1038/s41385-022-00535-6)
Supplement: Supplementary file 1 — Supplementary Information [file 41385_2022_535_MOESM1_ESM.pdf]

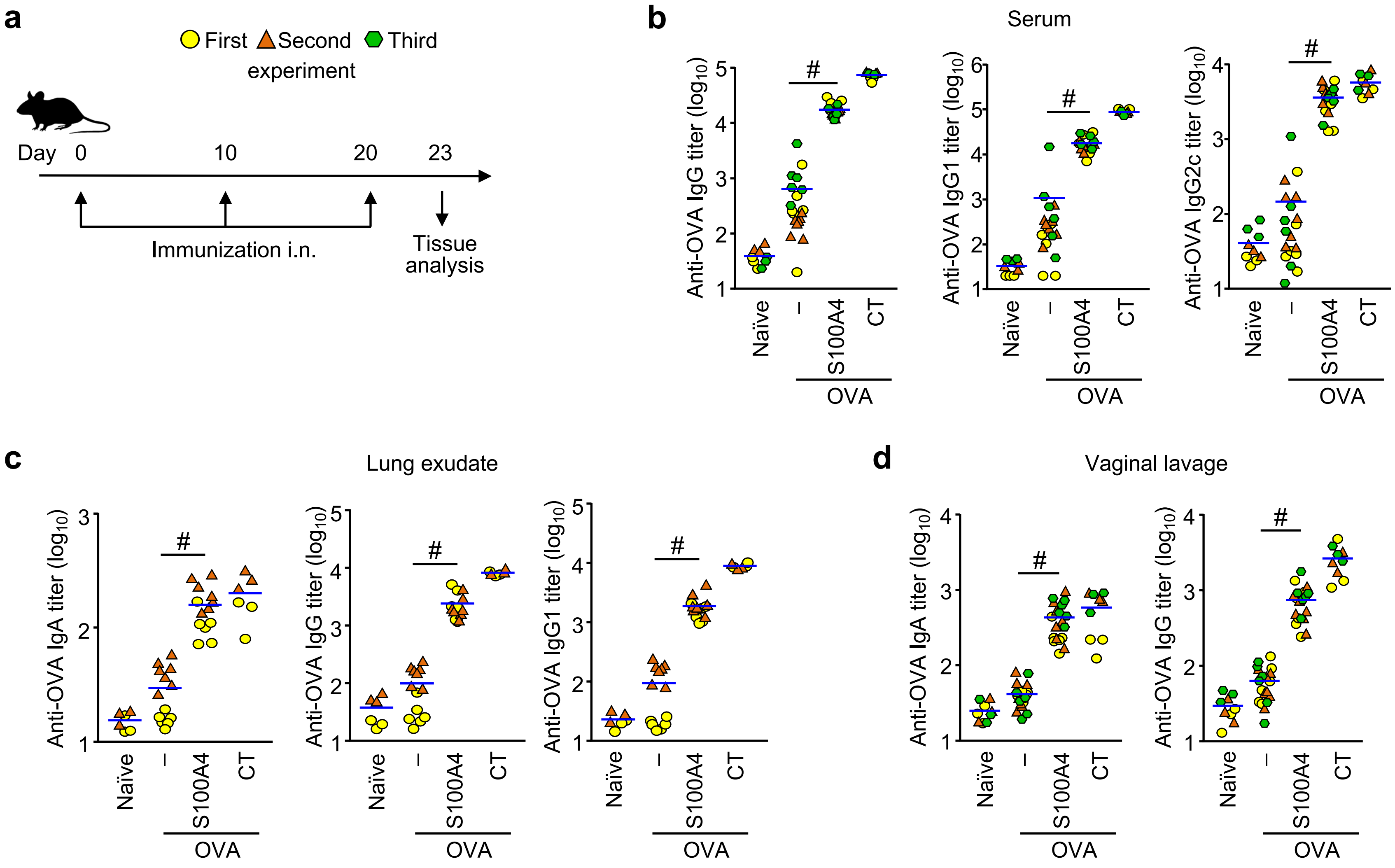

Supplementary Fig. 1 Intranasal immunization adjuvanted with S100A4 provokes greater humoral immune responses. Mice were immunized three times intranasally (i.n.) with ovalbumin (OVA; 10 μg) alone, or admixed to S100A4 (20 μg) or cholera toxin (CT; 1 μg) at a 10-day interval. Unmanipulated naïve mice were included for baseline control. Tissues were collected three days after the last immunization (**a**). Various classes of OVA-specific antibody levels in serum (**b**), lung exudates (**c**), and vaginal lavage (**d**) were analyzed by ELISA. Each dot represents data from an individual mouse and blue lines indicate the average values. Symbols of the same colour represent data from one experiment. Data from three (**b, d**) or two (**c**) experiments were pooled. #*P* < 0.0001 by Mann-Whitney *U* test.

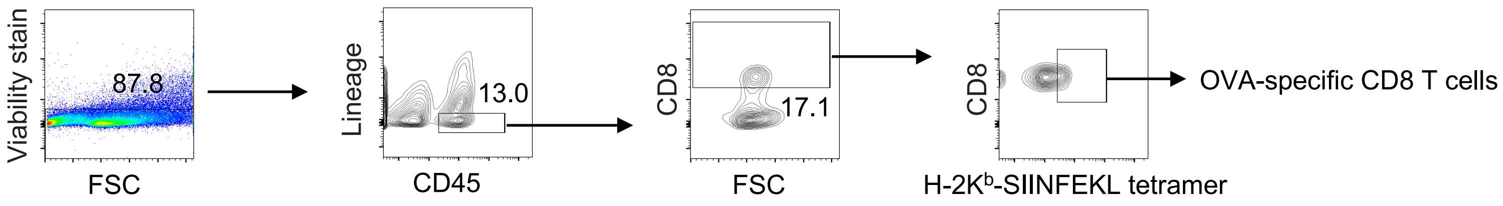

Supplementary Fig. 2 Gating strategies for lung CD8 T cell analysis are indicated. Mice were treated as explained in Figure 1a. Lung tissues were harvested 196 days after the last immunization for flow cytometric analysis. A group of antibodies (Ter-119, GR-1, CD11b, CD11c and CD19) were used to exclude cells of unwanted lineages. Arrows indicate gating strategies in flow cytometric analysis. Numbers adjacent to outlined areas indicate percent cells in each gate. Shown are an example of a random lung sample. The gating strategy also applies to the investigation of the lung, lymph nodes and spleen at 10 days after the last dose of immunization. Gating was determined based on naïve mouse controls.

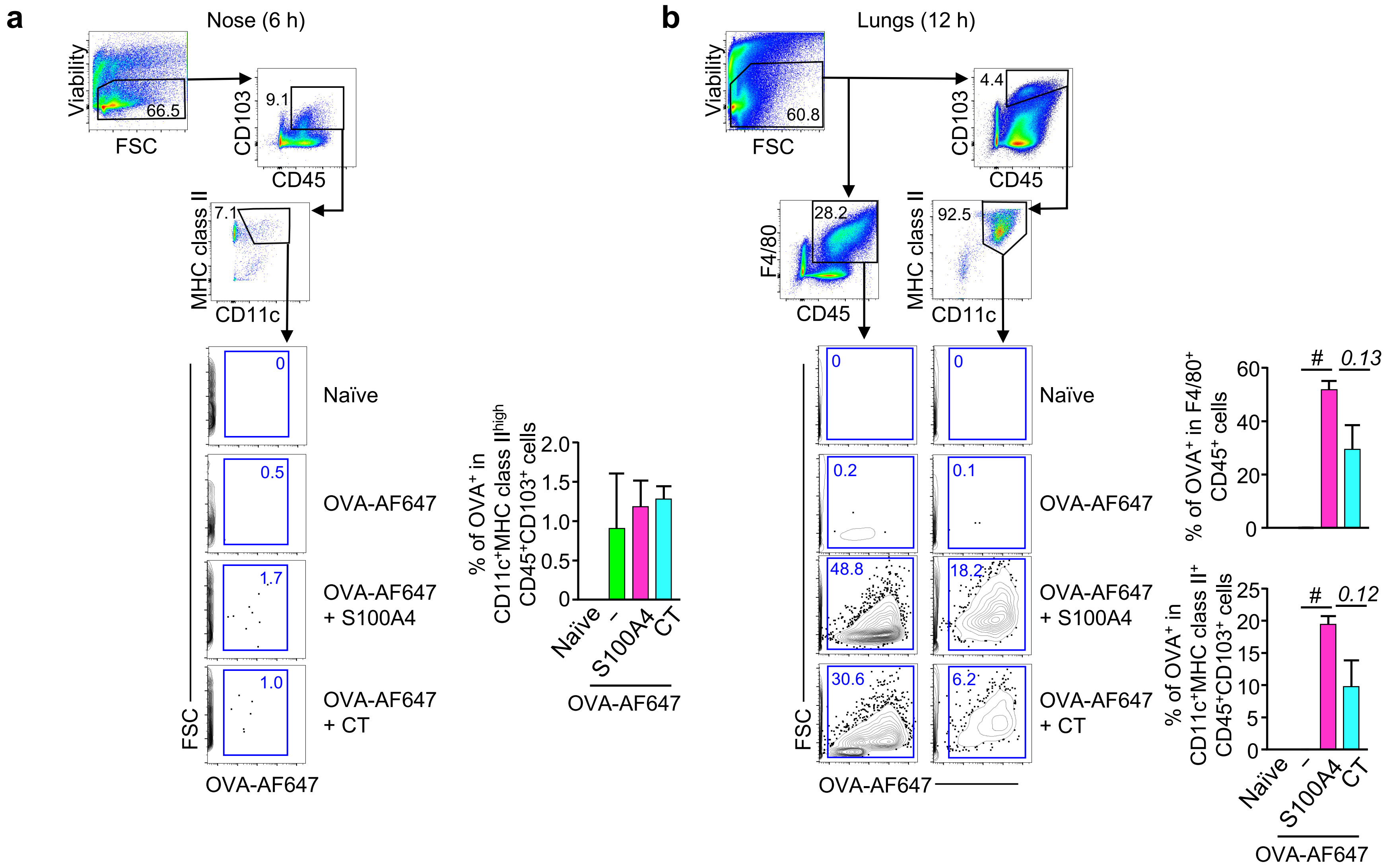

Supplementary Fig. 3 Further analysis of tissue transport of antigen delivered intranasally. Mice received a single intranasal delivery of Alexa Fluor 647-conjugated OVA (OVA-AF647; 10 μg) in the absence or presence of S100A4 (20 μg) or cholera toxin (CT; 1 μg). Unmanipulated naïve mice were included for background control. Mice were euthanized at 6 h **(a)** or 12 h **(b)** after OVA administration. Nasal tissues **(a)** and lungs **(b)** were harvested for flow cytometric analysis. Representative contour plots indicating the biodistribution of OVA-AF647 in one representative mouse out of four in each treatment group are shown. Numbers in or adjacent to outlined areas indicate percent cells in each gate. Data are expressed as mean + s.e.m. of four biological replicates. #*P* < 0.0001 or the exact *P*-values (italic numbers) are indicated by unpaired *t* test.

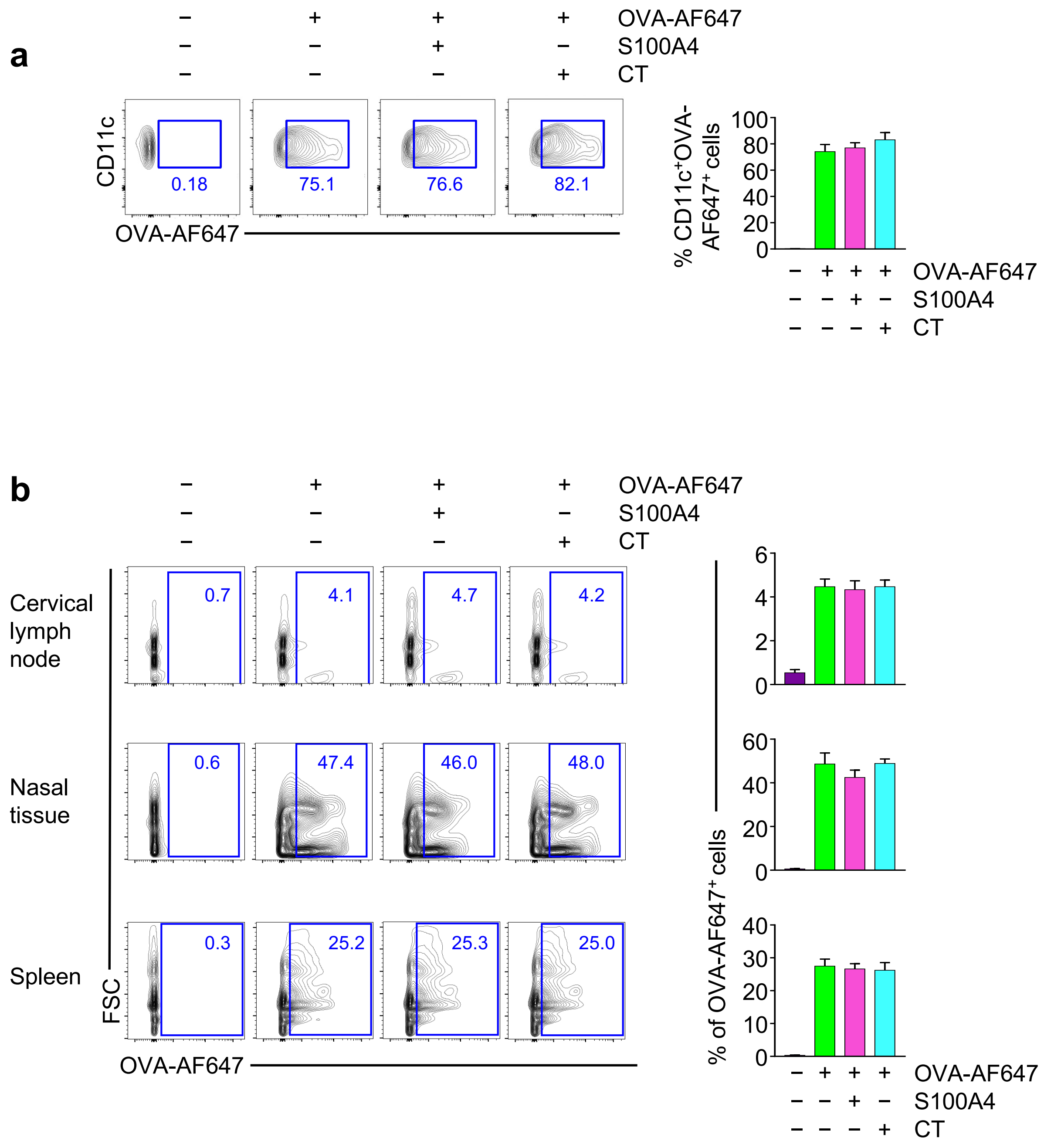

Supplementary Fig. 4 S100A4 does not promote antigen uptake by *in vitro* cultured dendritic cells and tissue cells. Bone marrow-derived dendritic cells (BMDCs) (**a**) and mouse *ex vivo* cells from various tissues as indicated (**b**) were incubated overnight with or without Alexa Fluor 647-conjugated ovalbumin (OVA-AF647; 10 μg/ml), in the presence or absence of S100A4 (1 μg/ml) or cholera toxin (CT; 0.1 μg/ml), followed by flow cytometric analysis of the uptake of OVA-AF647. Representative flow cytometry contour plots (left panels) and quantification of the data (mean + s.e.m.) pooled from three mice (right panels) are shown. Numbers in or adjacent to outlined areas indicate percent cells in each gate. Shown is one of three similar experiments.

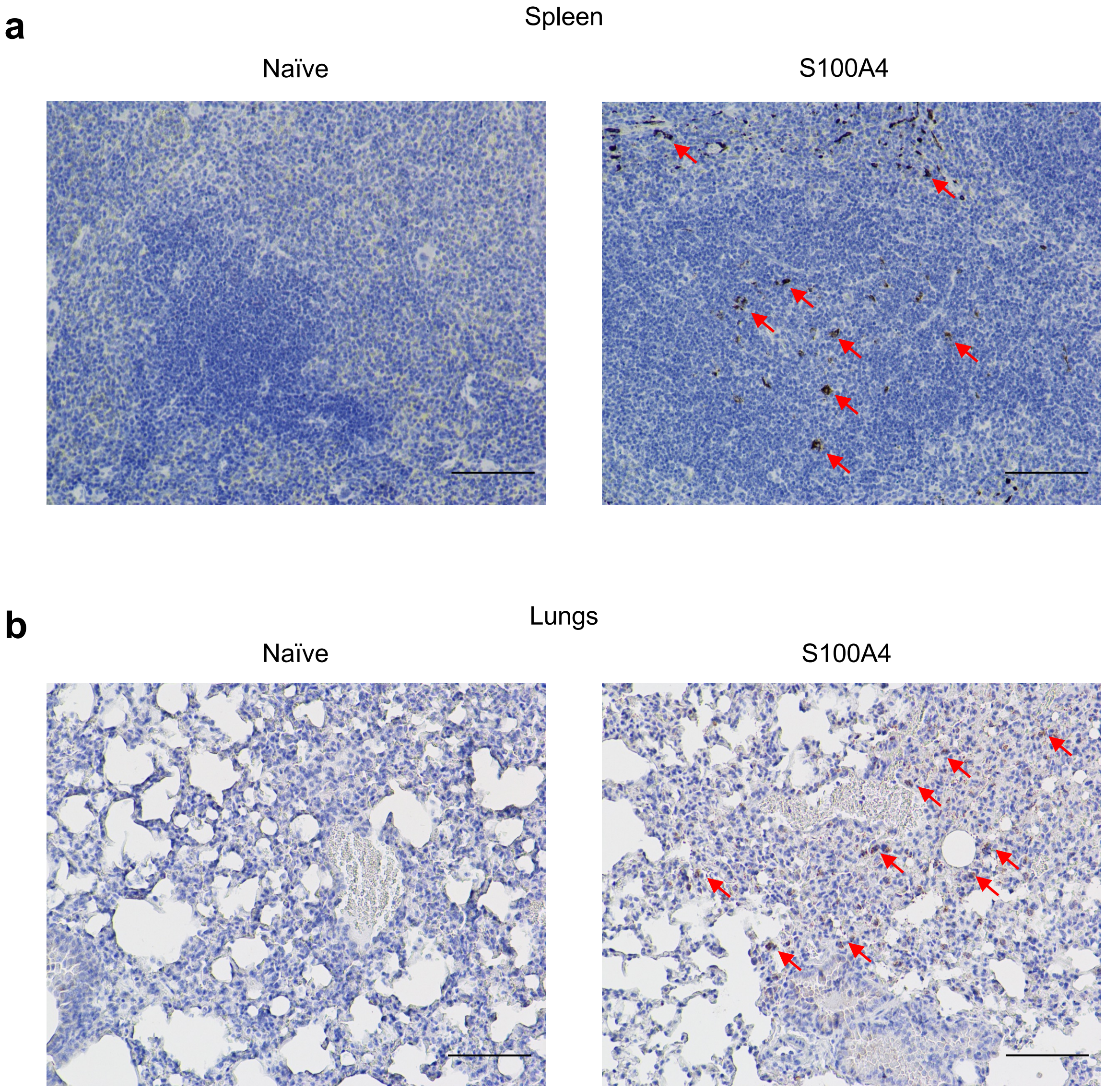

Supplementary Fig. 5 S100A4 migrates to the spleen and lung tissues following intranasal administration. Mice were intranasally administered with recombinant His-tagged S100A4 (20 μg). Unmanipulated naive mice were included for background control. Spleen (**a**) and lungs (**b**) were collected 24 h later, followed by sectioning and immunohistochemistry staining for the His-tag. Arrows indicate the His-tagged S100A4. Scale bar, 100 μm.

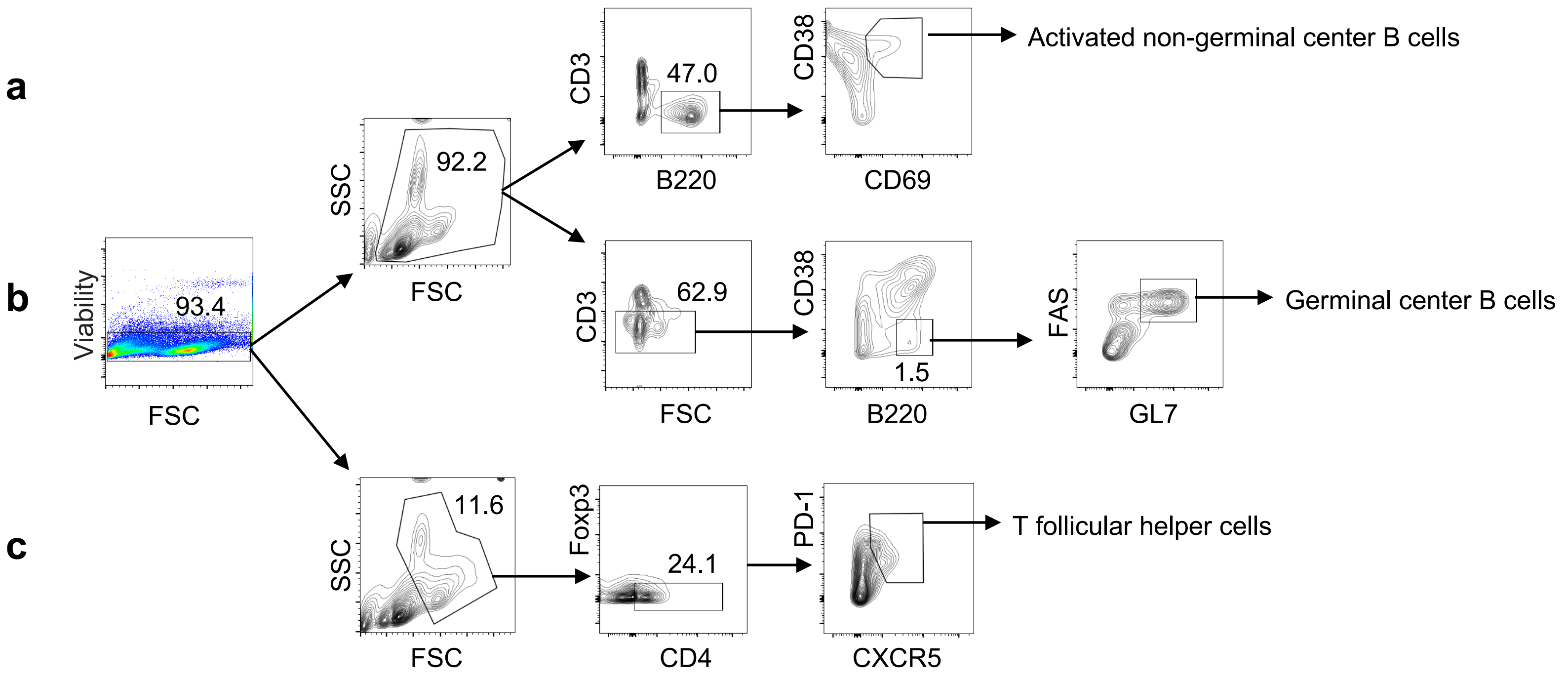

Supplementary Fig. 6 Gating strategies for splenic and lymph node cell analysis are shown. Mice were treated as in supplementary fig.1a. Spleens and cervical lymph nodes were harvested three days after the last immunization for flow cytometric analysis. Activated non-germinal center B cells were identified as $CD69^{+}CD38^{+}B220^{+}CD3^{-}$ cells (**a**). Germinal centre B cells were identified as $GL7^{+}FAS^{+}B220^{+}CD3^{-}CD38^{-}$ cells (**b**). T follicular helper cells were identified as $PD\text{-}1^{+}CXCR5^{+}Foxp3^{-}CD4^{+}$ cells (**c**). Arrows indicate gating strategies in flow cytometric analysis. Numbers in or adjacent to outlined areas indicate percent cells in each gate. Shown are examples of a random spleen sample. The gating strategies and the contour patterns for lymph node analysis are similar.

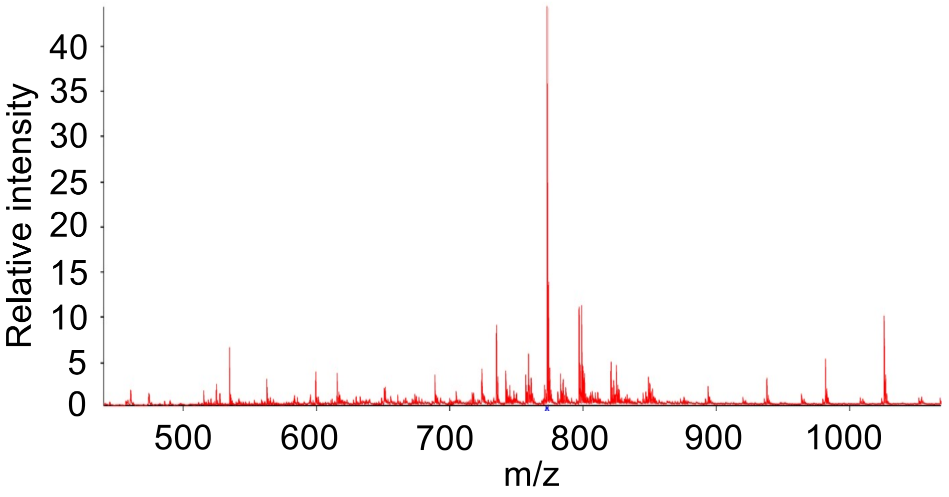

Supplementary Fig. 7 The mass spectra of all the lipids detected by MALDI-TOF are shown. Mice received three intranasal immunizations as explained in Supplementary Figure 1A. Spleens were collected three days after the last immunization and sectioned for MALDI-TOF analysis. A total of 348 lipids, including fatty acyls, glycerolipids, glycerophospholipids, polyketides, prenol lipids, and sterol lipids, were identified in the mouse spleens. About 83% of the total lipids were glycerolipids and glycerophospholipids. Shown is one example of a sample.

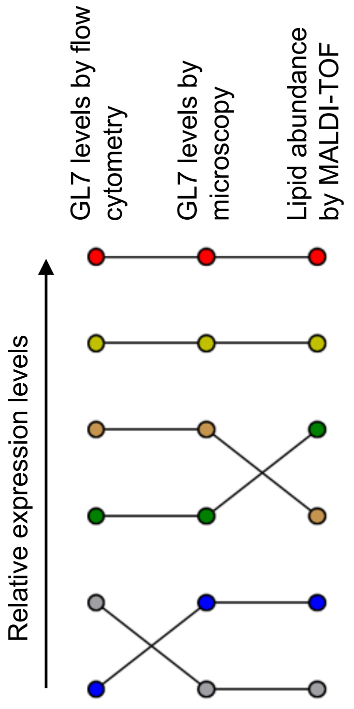

Supplementary Fig. 8 Spleen germinal center activities are consistent using different measurement techniques. Raw data displayed in Figure 4 and Figure 5 showing the expression levels of GL7 measured using flow cytometry or microscopy, as well as lipid abundance measured using MALDI-TOF, were processed and the expression levels of each (coloured dot) of the readouts were ranked at the single mouse level. Each line represents an individual mouse.

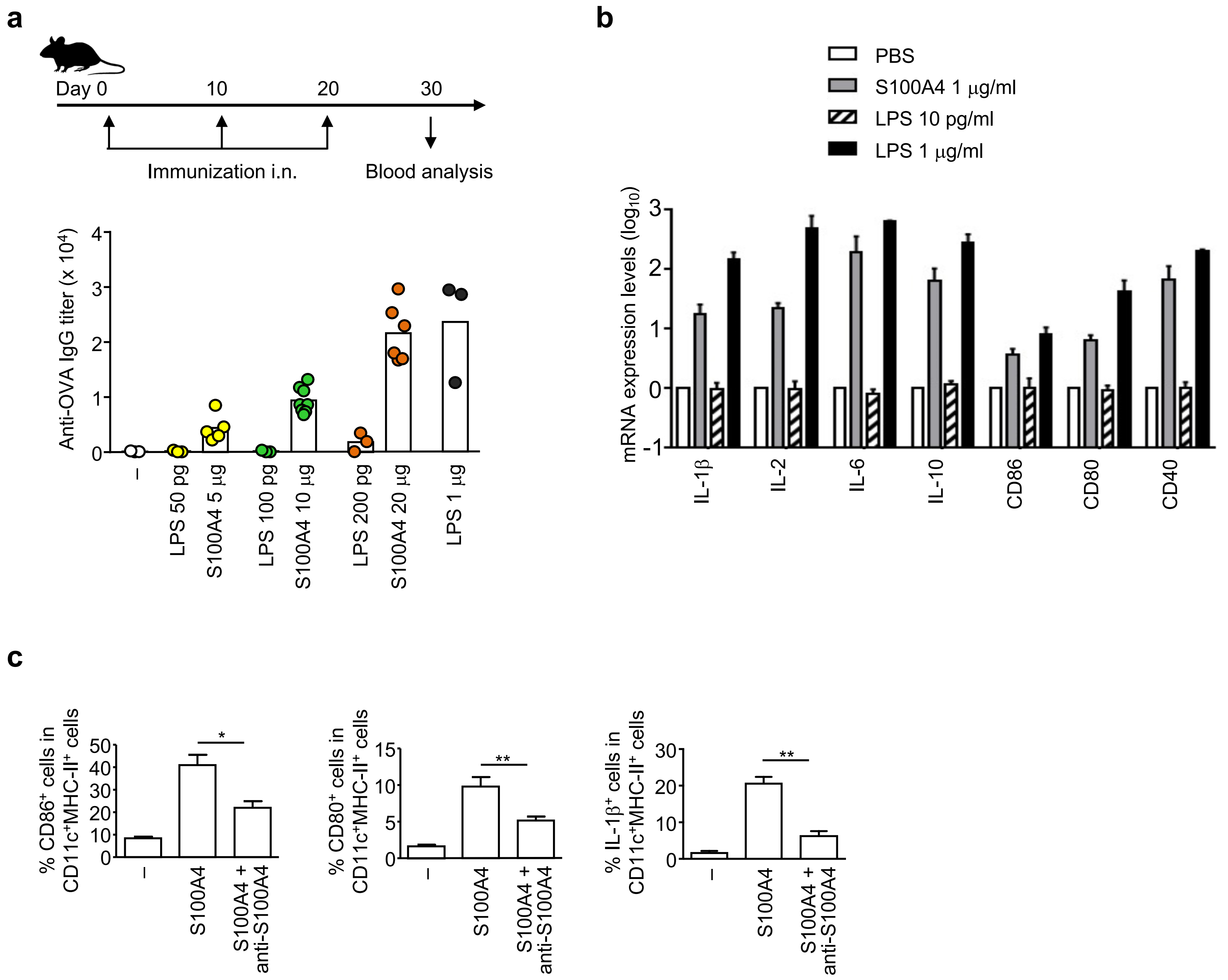

Supplementary Fig. 9 The adjuvant activity of S100A4 is not dependent on the residual amount of contaminating LPS. a Mice were immunized intranasally (i.n.) three times at a 10-day interval with ovalbumin (OVA; 10 μg) alone, OVA together with various amounts of S100A4 or with the corresponding residual LPS amounts present in each S100A4 dose. Serum was collected 10 days after the last immunization. OVA-specific total IgG levels were analysed by ELISA. Columns close to each other represent the pair that received an identical amount of LPS (exogenously added versus residual contamination in S100A4). **b** Bone marrow-derived dendritic cells (BMDCs) were incubated with S100A4 or the corresponding residual LPS amount present as indicated for 3 h and levels of mRNA expression of a panel of cytokines that could be augmented by LPS were assessed using quantitative reverse transcription PCR. Gene expression was normalized using GAPDH as the calibrator gene. **c** BMDCs were incubated for one day in the absence or presence of S100A4, or S100A4 pre-mixed with an anti-S100A4 antibody. The frequencies of activated BMDC with enhanced expression of CD86, CD80 or IL-1β were measured using flow cytometry. Each dot represents data from an individual mouse and dots of the same colour indicate an identical amount of LPS the mouse received; columns indicate the average values (**a**), or data are expressed as mean + s.e.m. of three biological replicates (**b**) or three separate experiments (**c**). *$P < 0.05$; **$P < 0.01$ by Mann-Whitney *U* test.

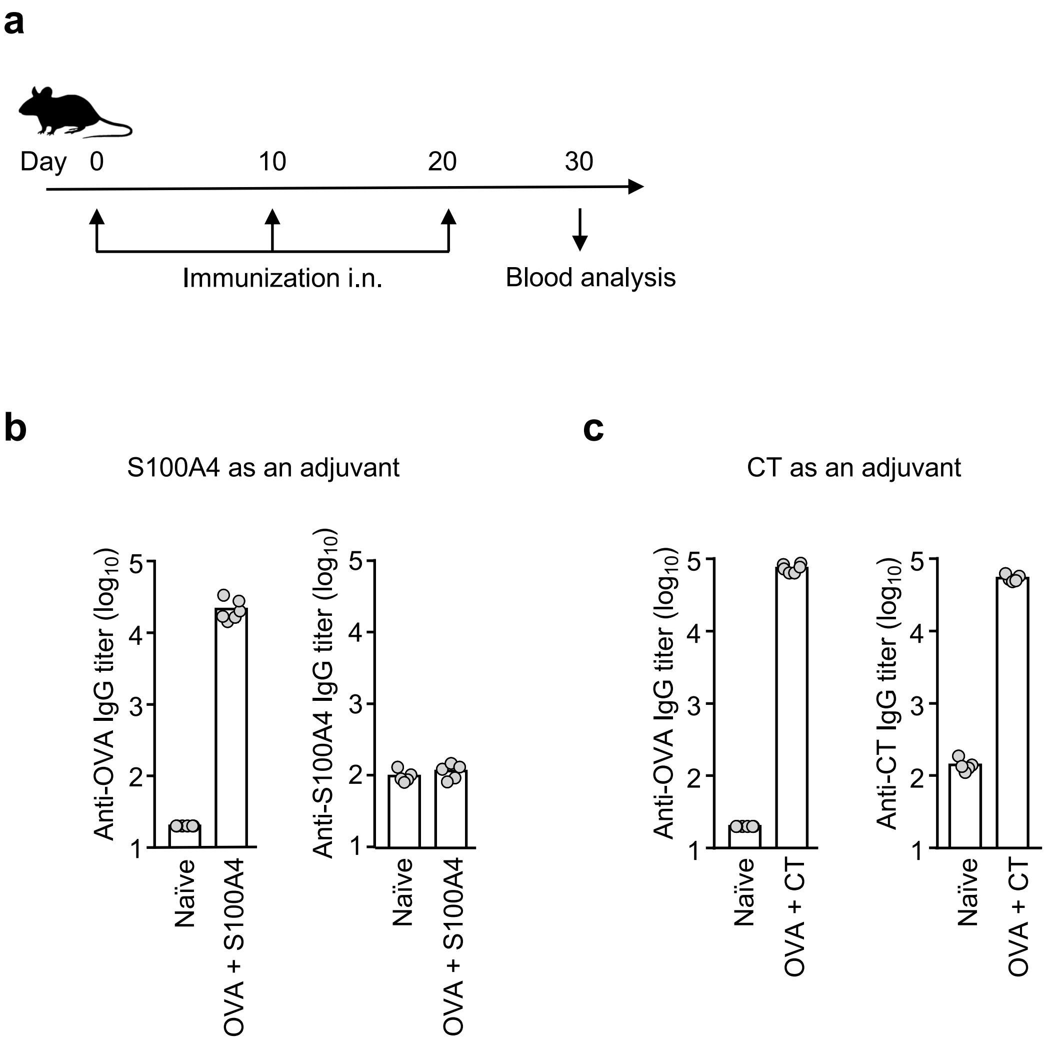

Supplementary Fig. 10 S100A4 does not induce anti-S100A4 antibodies after intranasal immunization. Mice were immunized three times intranasally (i.n.) with ovalbumin (OVA; 10 μg) alone or admixed to S100A4 (20 μg) or cholera toxin (CT; 1 μg) at a 10-day interval. Unmanipulated naïve mice were included for baseline control. Blood was collected 10 days after the last immunization **(a)**. Anti-OVA IgG antibody levels were determined for all the mice **(b, c)**. In addition, anti-S100A4 and anti-CT IgG antibody levels were determined for those mice which received S100A4 **(b)** or CT **(c)**, respectively. Antibodies were analyzed by ELISA. Each dot represents data from an individual mouse and columns indicate the average values.

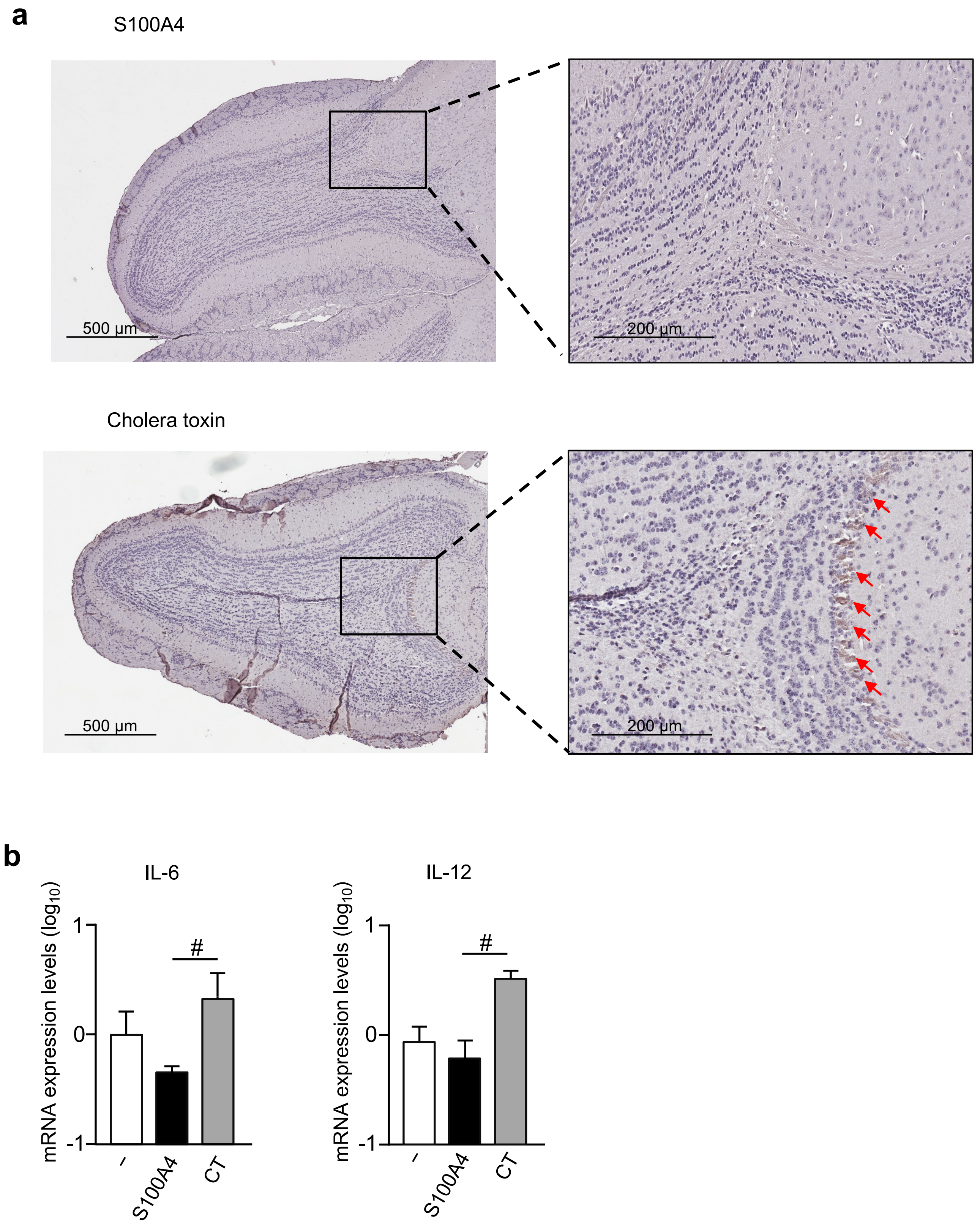

Supplementary Fig. 11 S100A4 does not induce olfactory bulb inflammation following intranasal administration. a Mice were intranasally administered with 20 μg S100A4 or 2 μg cholera toxin. The olfactory bulb was collected 24 h later, followed by sectioning and immunohistochemistry staining using an anti-CD45 antibody. Arrows indicate $CD45^+$ leukocytes. The boxed areas are magnified for clearer views. **b** Mice were immunized intranasally three times at a 10-day interval adjuvanted with or without S100A4 or cholera toxin (CT). Olfactory bulb was collected 10 days after the last immunization. IL-6 and IL-12 mRNA expression levels were analyzed by quantitative reverse transcription PCR. Gene expression was normalized using GAPDH as the calibrator gene. Data are expressed as mean + s.e.m. of seven biological replicates. #*P* = 0.0082 by Mann-Whitney *U* test
